# “*Tertius gaudens*”: germplasm exchange networks and agroecological knowledge among home gardeners in the Iberian Peninsula

**DOI:** 10.1186/1746-4269-9-53

**Published:** 2013-07-24

**Authors:** Victoria Reyes-García, José Luis Molina, Laura Calvet-Mir, Laura Aceituno-Mata, Juan J Lastra, Ricardo Ontillera, Montse Parada, Manuel Pardo-de-Santayana, Montse Rigat, Joan Vallès, Teresa Garnatje

**Affiliations:** 1 ICREA and Institut de Ciència i Tecnologia Ambientals, Universitat Autònoma de Barcelona, 08193 Bellaterra, Barcelona, Spain; 2 Departament d’Antropologia, Universitat Autònoma de Barcelona, 08193 Bellaterra, Barcelona, Spain; 3 Institut de Ciència i Tecnologia Ambientals, Universitat Autònoma de Barcelona, 08193 Bellatera, Barcelona, Spain; 4 Departamento de Biología (Botánica), Universidad Autónoma de Madrid, C/ Darwin 2. Campus de Cantoblanco, 28049, Madrid, Spain; 5 Departamento de Biología de Organismos y Sistemas, Universidad de Oviedo. Campus del Cristo, 33071, Oviedo, Spain; 6 Laboratori de Botànica, Facultat de Farmàcia, Universitat de Barcelona, Av. Joan XXIII, s.n, 08028, Barcelona, Catalonia, Spain; 7 Institut Botànic de Barcelona (IBB-CSIC-ICUB), Passeig del Migdia s.n., Parc de Montjuïc, 08038, Barcelona, Catalonia, Spain

**Keywords:** Home gardens, Ethnobotany, Germplasm exchange, *In situ* conservation, Landraces, Social network analysis, Spain, Traditional ecological knowledge

## Abstract

**Background:**

The idea that knowledge flows through social networks is implicit in research on traditional knowledge, but researchers have paid scant attention to the role of social networks in shaping its distribution. We bridge those two bodies of research and investigate a) the structure of network of exchange of plant propagation material (germplasm) and b) the relation between a person’s centrality in such network and his/her agroecological knowledge.

**Methods:**

We study 10 networks of germplasm exchange (n = 363) in mountain regions of the Iberian Peninsula. Data were collected through participant observation, semi-structured interviews, and a survey.

**Results:**

The networks display some structural characteristics (i.e., decentralization, presence of external actors) that could enhance the flow of knowledge and germplasm but also some characteristics that do not favor such flow (i.e., low density and fragmentation). We also find that a measure that captures the number of contacts of an individual in the germplasm exchange network is associated with the person’s agroecological knowledge.

**Conclusion:**

Our findings highlight the importance of social relations in the construction of traditional knowledge.

## Background

Local communities often possess a detailed knowledge of their resources [1,2] that potentially provides a valuable management base [3,4] and a source of resilience to deal with change [5]. Because of the potential of traditional ecological knowledge, *sensu* Berkes *et al.*[6], and associated practices to sustain the natural base for livelihoods, researchers have tried to understand the pathways for the acquisition, maintenance, erosion, and spread of this type of knowledge. Previous research has noticed that traditional ecological knowledge is not uniformly distributed among resource users, and consequently, researchers have studied the sociodemographic characteristics that pattern intracultural distribution of knowledge. Researchers have found that age, sex, education, kinship, place of residency, social status, level of acculturation, and level of integration into the market economy, among others, correlate with intracultural variation in traditional ecological knowledge [7-10].

Researchers have based the systematic analysis of the pathways through which traditional ecological knowledge is transmitted on the seminal work of Cavalli-Sforza and colleagues [11]. Based on generational differences and social relations between actors, this work considers three main pathways for the transmission of cultural knowledge: 1) vertical transmission, when information flows across individuals from different generations related through kinship [11,12], 2) horizontal transmission, when information is transmitted between any two individuals of the same generation [13], and 3) oblique transmission, when information flows across individuals from different generations but not related through kinship [11,14]. Recent research shows that the influence of each pathway changes across a person’s lifecycle [15].

While the idea that cultural knowledge flows through social relations is implicit in this line of thought, researchers have not engaged in a systematic analysis of the relations between the structure of social networks and the distribution of traditional ecological knowledge. This research gap is especially surprising given the well established finding from research on social networks highlighting that a node’s position in the network is a measure of the degree and type of information the node holds [12-14]. For example, in organizational settings, Brass and Burkhardt [15] have shown that the ability of a firm to capture knowledge is influenced by the position of the firm in the network. Similarly, Burt [12] shows how structural holes, i.e., the ability of an individual to bridge the gap among otherwise disconnected social groupings, bring new ideas to individuals because of their ability for assessing and comparing the different social groupings in which they are active.

The scant systematic research on the role of social relations on the transmission of traditional ecological knowledge has highlighted that information sharing among resource managers is based on trust and mostly occurs through kinship, friendship [16], or other social relations such as occupation [17] or ethnic background [18]. But we know little about how structural characteristics that have proven central in the transmission of other types of information affect the transmission of traditional ecological knowledge. In this article we bridge research on the distribution of traditional ecological knowledge and research on social networks to investigate the relation between a person’s position in a network and her/his agroecological knowledge. We start by analyzing the structure of plant propagation material exchange networks and we then assess the association between a gardeners’ centrality in such networks and their agroecological knowledge. Research was conducted among home garden tenders in mountain regions of the Iberian Peninsula.

Our study focuses on networks of exchange of plant propagation material, mainly seeds, but also seedlings, bulbs, tubers, cuttings, suckers (hereafter germplasm exchange networks). We studied home gardens because they are considered *in situ* repositories of genetic diversity [19] and legacies of traditional gardening practices. Furthermore, both germplasm and knowledge are transmitted over generations [20,21].

Based on Berkes *et al.*[6] and Armitage [22], we use the term “agroecological knowledge” to refer to the cumulative body of knowledges, practices, and beliefs related to agronomic practices evolving by adaptive processes and handed down through generations by cultural transmission. We follow Calvet-Mir *et al.*[23] and use the term “landrace” to refer to annual and biennial crops that have been continuously reproduced by gardeners during more than one generation (30 years or more, or 60 in the case of perennial crops and crops with vegetative reproduction) in the geographic area of study.

Researchers have previously described networks of germplasm exchange in different landscapes and socioeconomic conditions [24] highlighting the contribution of those exchanges to the conservation of agrobiodiversity in farmers’ fields [25-28]. For example, Boster [7] analyzed the social processes through which manioc (*Manihot esculenta* Crantz) stem cuttings are transmitted, sustained, and enhanced through networks of female linked by kinship ties; in a study in Peru, Ban and Coomes [26] found that home gardens agrobiodiversity is strongly tied to the number of seedlings and seed exchanges done by the gardeners; and Ellen and Platten [29] described germplasm circulation (or “the social life of seeds”) in British allotments. But, with some exceptions (see [30,31]), those studies have not analyzed the link between the person’s structural position in the germplasm exchange network and his/her agroecological knowledge.

## Methods

### Study sites

We used a multicommunity approach and analyzed 10 independent networks placed in the Catalan Pyrenees (5), central Asturias (1), and Sierra Norte, Madrid (4). The three areas are in mountain regions, where the prevalence of slopes makes intensive agriculture difficult. Despite their linguistic and cultural differences since the 1960s, the three areas have suffered similar changes in the commercial agricultural sector resulting either in the concentration of agricultural activities in the more productive lands or in their abandonment by shifting to other activities (mining, construction, services) [32]. However, in the three areas home gardens persist nowadays as the most characteristic and widespread form of agriculture [23,33,34] providing a non-negligible financial gross value as well as social benefits [35,36].

### Sample

Our sampling strategy proceeded in two steps. On the first scooping step, we selected a range of villages representing the environmental and socioeconomic variability of the areas and interviewed between 20% and 100% of the households (depending on the total number of active home gardens) [36]. This preliminary work served us to identify 10 germplasm exchange networks, defined as a group of gardeners potentially related by the exchange of seeds or other plant propagation material. On the second step, we interviewed all the primary tenders (i.e., the person who reportedly made most of the decisions and carried out most of the work on the home garden) in the 10 selected networks. Our total sample includes 363 individuals in 10 independent networks across the three study areas: eight of the networks correspond to villages and two (Vall Fosca and Valle del Cordal) to valleys with several small villages closely related one to each other (see Table 1 for a breakdown of the sample site by network).

**Table 1:** Structure of ten seed exchange networks in the Iberian Peninsula

		**[**[1]**]**	**[**[2]**]**	**[**[3]**]**	**[**[4]**]**
**Area**	**Network name (n)**	**# Nodes^ (Gardeners)**	**Density**	**Components**	**Network centralization (%)**
Asturias	Valle del Cordal (n = 56)	118 ( = 40 + 56 + 22)	0.017	6	8.09
Catalan Pyrenees	Llanars (n = 21)	50 ( = 0 + 21 + 29)	0.019	15*	6.68
	Llançà (n = 15)	24 ( = 5 + 15 + 4)	0.052	11	21.54
	Maçanet de Cabrenys (n = 31)	34 ( = 0 + 31 + 3)	0.043	14*	12.69
	Molló (n = 34)	42 ( = 0 + 34 + 8)	0.023	16*	7.99
	Vall Fosca (n = 55)	111 ( = 14 + 55 + 42)	0.018	5	4.91
Sierra Norte de Madrid	Canencia (n = 23)	33 ( = 0 + 23 + 10)	0.037	7	6.94
	Montejo de la Sierra (n = 27)	65 ( = 16 + 27 + 22)	0.032	3	16.39
	Patones (n = 40)	114 ( = 15 + 40 + 59)	0.025	7	12.01
	Valdemanco (n = 61)	108 ( = 22 + 61 + 25)	0.020	4	7.14

^ # Nodes or Gardeners = (Nodes outside research area + nodes with complete data for multivariate analysis + nodes with incomplete data or not interviewed). * Less than 50% of the nodes belong to the main network.

### Methods of data collection

A multidisciplinary team of social and natural scientists collected data during April 2008-October 2009 using participant observation, semi-structured interviews, garden inventories, and a survey.

#### Participant observation

Six researchers had a continuous presence in the study sites for about a year (some of them are residents to the study areas). The rest of the team conducted shorter visits to some of the sites. Participant observation allowed the understanding of the different activities and tasks around gardening by giving us ample opportunities -other than during the formal interviews- to interact with gardeners and to discuss garden’s progress and other issues. The results of the ethnographic research have been presented in previous works [33-35,37].

#### Semi-structured interviews

We also carried out semi-structured interviews with more than 90 ‘local experts’ (about 30 per study area), defined here as local inhabitants with long-term experience with traditional management of home gardens in the area [38]. During semi-structured interviews we asked about the practice of exchanging seeds and other germplasm and its purpose, persistence, and usefulness. We also discussed traditional management of home gardens and changes on management techniques over the last decades.

#### Garden inventories

We visited each garden in the sample and requested the self-reported main tender to accompany us. We asked the tender to identify all the cultivated plants present in the garden. We took a picture and recorded the local name and the main use (i.e., edible, medicinal, ornamental) of each species. We noted the species that matched our definition of landrace. We determined the species (scientific names) mainly in the field, but we took vouchers of species that could not be easily identified in the field for laboratory identification. Vouchers have been deposited in the herbarium of the Centre de Documentació de Biodiversitat Vegetal, Universitat de Barcelona (BCN), in the herbarium of the Departamento de Biología de Organismos y Sistemas, Universidad de Oviedo (FCO), or in the herbarium of the Real Jardín Botánico, CSIC, in Madrid (MA).

#### Survey

We based the construction of the survey in information collected with participant observation, semi-structured interviews, and garden inventory. We conducted a survey with all the primary garden keepers. The survey had three sections. In the first section, we asked about sociodemographic characteristics of the person answering the survey (age, sex, maximum education level, years gardening, and length of residency). In the second section, we asked gardeners about their germplasm exchange network using a name-generator technique [39]. Specifically, we asked, “Could you please list the name of all the people to whom you have ever given seeds or any other type of propagule?” Once the person stopped providing names, we asked “Could you please list the name of all the people who has ever given you seeds or any other type of propagule?” For each name listed, we also asked the respondent to provide the place of residence.

To evaluate gardeners’ agroecological knowledge, the last section of the survey consisted of a knowledge test. Because species and practices vary from one site to another, we constructed site-specific knowledge tests (i.e., we used 10 different tests, one for each site). To ensure comparability of results across sites, we constructed the 10 tests following the same protocol and with the same structure, in each case using information from semi-structured interviews and garden inventories.

Our knowledge tests included a section on landraces and a section on traditional management practices. To capture variability in knowledge and to keep the survey in a reasonable length, we used information from garden inventories (i.e., frequency of appearance) participant observations to select one well known, one relatively known, and one rare landrace in each site. For each landrace we requested gardeners a) to provide the folk name of the species (by identifying seeds and pictures) (*SpName*), to report whether they b) were growing the landrace at the time of the interview (*SpPlant*), c) had grown it during previous years (*SpPlantPast*), d) had it in storage (*SpSeed*), and e) to answer a question on landrace management (*SpManag*) and f) a question on landrace use (*SpConsum*). The section on traditional management practices included questions on whether the gardener had ever applied specific traditional management practices in the garden (*TradManagPast*) and four questions on whether the gardener used the same practices at the moment of the interview (*TradManagNow)*. Questions on landrace management and use and questions on the traditional management practices were selected from information collected during semi-structured interviews with locally recognized experts. Table 2 presents one of the knowledge test used as an example.

**Table 2:** Example of knowledge test (from the Llançà network)

**Alone**	**Did the person answer all the survey without help**	**YES**	**NO**
**Traditional common**	***Brassica oleracea*var.*oleracea***		
Sp1Name	What is the name of this plant?	(text)	
Sp1Plant	Have you planted it in your garden this year?	YES	NO
Sp1Plantpast	Have you planted it in previous years?	YES	NO
Sp1Seed	Did you keep seeds from last year?	YES	NO
Sp1Manag	What is the best moment to plant this species?	(text)	
Sp1Consum	How do you know when is ready for human consumption?	(text)	
**Traditional intermediate**	***Brassica napus***		
Sp2Name	What is the name of this plant?	(text)	
Sp2Plant	Have you planted it in your garden this year?	YES	NO
Sp2Plantpast	Have you planted it in previous years?	YES	NO
Sp2Seed	Did you keep seeds from last year?	YES	NO
Sp2Maneg	What is the best moment to plant this species?	(text)	
Sp2Consum	How do you know when is ready for human consumption?	(text)	
**Traditional rare**	***Cynara cardunculus***		
Sp3Name	What is the name of this plant?	(text)	
Sp3Plant	Have you planted it in your garden this year?	YES	NO
Sp3Plantpast	Have you planted it in previous years?	YES	NO
Sp3Seed	Do you try to keep it in your garden?	YES	NO
Sp3Maneg	What is the edible part?	(text)	
Sp3Consum	What are its uses?	(text)	
**Management**			
TradManag1Now	Do you use moon cycles to plant?	YES	NO
TradManag1Past	Did you use to do it before?	YES	NO
TradManag2Now	Have you done tomatoes preserves this year?	YES	NO
TradManag2Past	Did you use to do it before?	YES	NO
TradManag3Now	Do you prepare a seedbed of this plant?	YES	NO
TradManag3Past	Did you use to do it before?	YES	NO
TradManag4Now	Do you put ashes in the place prepared to plant garlic and onions?	YES	NO
TradManag4Past	Did you use to do it before?	YES	NO

### Methods of data analysis

#### Social network analysis

We used social network analysis a) to examine the structure of the germplasm exchange networks and b) to generate two variables that capture the structural position of an individual in the network [40-42]. Information was treated with UCInet6-Netdraw for Windows [43]. To explore the structure of the network of plant propagation exchange, we first added information on nominations as seed givers and seed receivers. Adding the two matrices we obtain a single enlarged matrix with the value “2” in the cells corresponding to reciprocal nominations (A mentions B as exchange partner and B mentions A as exchange partner as well), the value “1” for non-reciprocal nominations, and “0” for the rest (see [30] for a detailed explanation).

We then calculated four measures that describe the structure of a network: 1) *Size*, or number of gardeners (nodes) in the network, including gardeners in the research areas and gardeners outside them; 2) *Number of components* or connected sub-networks in which all gardeners are directly or indirectly in contact with each other; 3) *Density* or the proportion of existing links in the network relative to the maximum possible number of links (from 0 to 1); and 4) *Network centralization* or the tendency for a few gardeners to concentrate the existing links (expressed as a percentage).

We used the same data to generate two different variables that capture the structural position of an individual in the network: 1) *Weighted degree* or the sum of the number of nominations that a gardener received on other’s lists plus the number of nominations that the gardener made, and 2) *Broker* or the number of times a gardener (*ego)* lies on the shortest path between two other gardeners (*alters*) not connected among them. This variable captures the contribution of a gardener to minimize the distance between gardeners in the germplasm exchange network [43].

#### Construction of outcome variables

A person’s *agroecological knowledge* score was constructed using answers to the questions on the knowledge test, with a range from 0 to 26. We added a point to the *agroecological knowledge* score if the informant a) was able to provide the folk name of the landrace, b) was growing the landrace at the time of the interview, c) had grown the landrace during previous years, d) or had the landrace in storage. We added additional points if answers to questions on landrace management and use matched the information provided by local experts during semi-structured interviews. Last, we also added points to the score of *agroecological knowledge* for each of the traditional management practices the gardener reportedly applied (in the past or nowadays). Surveys were pre-tested with gardeners outside our sample.

### Statistical analysis

To estimate the association between a person’s agroecological knowledge and structural position in the germplasm exchange network, we ran a Poisson multivariate regression with (a) *agroecological knowledge* as outcome variable and (b) our two centrality measures (*degree* and *broker)* as explanatory variables while controlling for additional factors that research suggest affect the distribution of traditional ecological knowledge (i.e., age, sex, years gardening, schooling, and years of residency). Because networks vary in structure, before conducting the analysis, we normalized the two measures (by dividing by the mean) and used the normalized version. For the statistical analysis we used STATA 9 for Windows.

## Results

### Structure of the germplasm exchange networks

**Figure 1:**
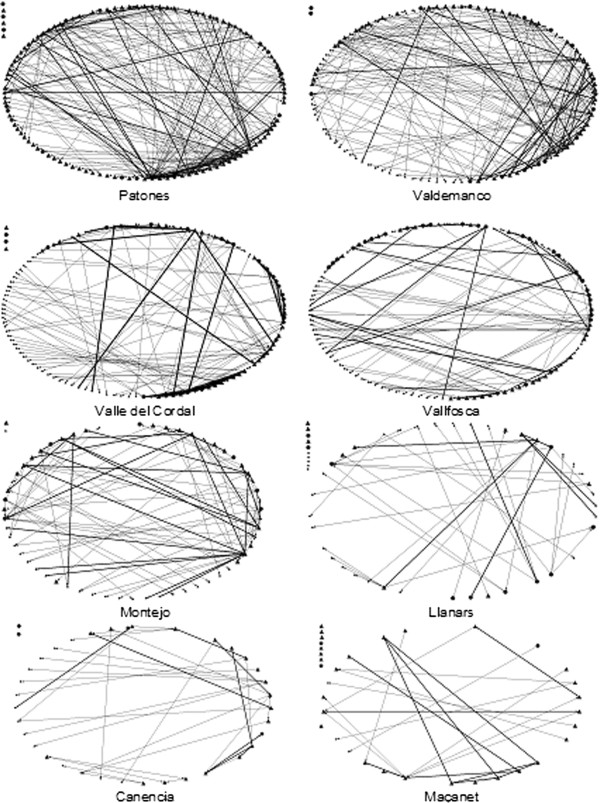
**Eight germplasm exchange networks in the Iberian peninsula, from more to less dense.** Note: Nodes are shaped by the sex of the gardener (triangle for men, circle for women, and a square for a local seed bank) and sized by the score of agroecological knowledge. Edges arrow represents the direction of the nomination and edges thickness the intensity of exchange.

Germplasm exchange networks varied in the number of gardeners that composed them (from 24 to 118) and in the proportion of gardeners from outside the research area (Table 1, Figure 1). Overall, the studied networks have low densities (Column 2) and are highly fragmented, i.e., have several sub-networks (Column 3). From a range from 0 to 1, the highest density level was around 0.05, indicating that there are few ties even between the gardeners that are actually connected on the same network.

Our data also suggest that gardeners that could potentially belong to a simgle germplasm exchange network were in fact organized in smaller groups. All the networks analyzed but two have five or more components or sub-networks, meaning that gardeners were actually organized in several disconnected networks (Column 3). Four networks had more than 10 sub-networks and one had as much as 16 (most of them isolated gardeners with no exchanges within the network). In three of the 10 networks, the largest component contains less than 50% of the gardeners that could potentially be linked. The network centralization indexes are also low (Column 4). Relative to a pure star network, where a single gardener would hold all connections to the rest (otherwise unconnected) gardeners, only four of the networks analyzed had a centralization index higher than 10%, and only one had a centralization index higher than 20%.

### Agroecological knowledge and network centrality

The average *agroecological knowledge* is close to the medium value (13), but displays large variation among people in the sample (mean = 13.7; SD = 5.37) (Table 3). The average values for the variables that measure *degree* were low (mean = 4.77; SD = 4.26; min = 0; max = 30), meaning that -on average- each person gave or received seeds and other propagules from 4.77 different people. The average value for the variable *broker* was of 9.9, meaning that on average, each gardener connects 9.9 pairs of otherwise unconnected gardeners. As the previous variable, the variable *broker* displays large variation (SD = 23.66), meaning that some gardeners had a much stronger centralizing role than others.

**Table 3:** Definition and summary statistics of variables used in regressions

**Variable**	**Definition**	**N**	**Mean**	**S.D.**
***I. Outcome variable:***				
*Agroecological knowledge*	Sum from scores on *landraces knowledge* and *traditional management practices* (from 0 to 26)	363	13.7	5.37
***II. Explanatory variables:***				
*Degree*	Number of nominations given plus number of nominations received by the person in the network of germplams seed exchange	363	4.77	4.26
*Broker*	Number of pairs connected through ego	363	9.9	23.66
***III. Control variables:***				
*Age*	Age of the person, in years	363	66.1	13.79
*Male*	Dummy variable that captures the sex of the person interviewed, 1 = male, 0 = female	363	0.68	0.46
*Years gardening*	Number of years the person has been gardening	363	44.7	24.26
*Egonetwork*	Size of the person’s networks	363	3.78	3.38
		**N**	**%**	
*Schooling*	No schooling	45	12.40	
	Primary school	176	48.48	
	Between primary school and university degree	117	32.23	
	University degree	25	6.89	
*Years of residency*	Never been resident	15	4.13	
	Between 1 and 5 years	8	2.20	
	Between 5 and 10 years	12	3.31	
	More than10 years	127	34.99	
	Always been resident	201	55.37	

Results from Table 4 suggest a positive association between *agroecological knowledge* and one of our two centrality measures: *broker*. Thus, gardeners who connect more pairs of otherwise unconnected gardeners (i.e., higher value in the variable *broker*) have higher *agroecological knowledge* than people with a lower value in the variable *broker*. The association is statistically significant at the 95% confidence interval. We do not find a statistically significant association between the *degree* of a person and his/her *agroecological knowledge*, which implies that the number of people with whom a gardener exchanges plant propagation material is not related in a statistically significant way to the person’s agroecological knowledge.

**Table 4:** Multivariate Poisson regression: agroecological knowledge and centrality in the network of germplasm exchange (n = 363)

	**Agroecological knowledge**
**Explanatory variables:**	
*Weighted degree* (normalized)	0.004 (0.009)
*Broker* (normalized)	0.279*** (0.099)
**Control variables:**	
*Age*	−0.005 (0.002)
*Male*	−0.084** (0.035)
*Years gardening*	0.007*** (0.001)
*Network size*	0.001 (0.010)
*Schooling* (ref. category no education)	
Primary school	−0.001 (0.048)
More than primary but without university degree	−0.047 (0.057)
With university degree	−0.095 (0.092)
*Residency duration* (ref category, never lived)	
Between 1 and 5 years	0.101 (0.146)
Between 5 and 10 years	0.240* (0.131)
More than10 years	0.275*** (0.093)
Always resident	0.320*** (0.093)
**R**^**2**^	0.18

Note: For definition of variables see Table 1. Robust standard errors in parenthesis. *, **, and *** significant at the 10%, 5% and 1% level. Regressions contain a set of dummy variables for the network of data collection and a constant (not shown).

We tested the robustness of the findings by running a set of variations of the model (Table 5). In our first two robustness models, we included additional control variables that might affect an individual’s agroecological knowledge and her centrality in the exchange network. In model [2] we included migratory status as control, as migrants might have different cultural background, which might increase the cost of acquiring the local agroecological knowledge [25,44]. In model [3] we included participation on social organizations in the area as engagement in collective action institutions can facilitate access to information and foster germplasm exchanges [25]. Model [4] resembles the core model, except that we ran the regression with the option clustering, and the network of seed exchange designated as the clustering variable. This option relaxes the assumption that individual observations are independent and requires only independence across clusters. This procedure adjusts for the fact that networks are the social units where gardeners exchange planting material and provides robust (and more conservative) estimates of variance around regression parameters. In model [5] we excluded the variables for the village of residency of gardeners. In model [6], we rescaled the value of the knowledge variable to improve comparability in knowledge scores collected with different questionnaires. Specifically, we calculated the deviation from the mean, or Z-score, of our measure of *agroecological knowledge* for each of our 10 networks. The results from our robustness analysis mostly confirm two findings of the core model. First, the *degree* of a person was not associated with his/her level of agroecological knowledge. Second, the value of the variable *broker* bears a positive and statistically significant association with the level of agroecological knowledge of a person in all the models.

**Table 5:** Robustness analysis

**Changes:**		***Degree***	***Broker***
[1]	Core model (as in Table 3)	0.004 (0.009)	0.279*** (0.099)
[2]	Controlling for the migratory status of the person	0.003 (0.009)	0.287*** (0.101)
[3]	Controlling for participation in organizations	0.001 (0.009)	0.300*** (0.099)
[4]	Clustering by seed exchange networks	0.004 (0.012)	0.279* (0.171)
[5]	Excluding dummies for village of residency	0.005 (0.009)	0.230** (0.096)
[6]	Using Z-score value for agroecological knowledge score (and Ordinary Least Square regression model)	0.025 (0.024)	0.737** (0.316)

Note: Regressions resemble the model in Table 3, except for the changes specified in the column “Changes”. Robust standard errors in parenthesis. *, **, and *** significant at the 10%, 5% and 1% level.

## Discussion

### Structure of germplasm exchange networks

The analyzed networks display three common factors: low density of interactions (indicating that there are few exchanges between the actors), fragmentation (meaning that the actors are joint in different disconnected groups), and decentralization (implying that there are not main actors that agglutinate the majority of exchanges). Low interaction density of germplasm exchange dovetails with research highlighting that, despite their importance for *in situ* agrobiodiversity conservation [19], exchanges are not the primary seed source. In previous research [21], it has been reported that the primary propagules sources for most crops were from the farmer’s own harvest and the market place. Our own previous research suggests the same pattern in the area. Thus, in Sierra Norte, 45% of the propagules are from commercial origin and 32% from the own farmer [33]. So, a potential explanation of the low density of exchange of germplasm in our study areas lays in the presence of a formal seed supply system and in the prevalence of commercial varieties. In the study sites seed saving has increasingly been restricted to a smaller number of crops not available in the market. Several of our oldest informants mentioned that seed saving and exchanging was more common in the past, when there were no markets for seeds, but as seeds and plantlets for most varieties are conveniently available at the local markets, the number of exchanges has decreased. The extra work required to prepare the seedbed and the degeneration of seeds are disincentives to maintain a local seed system [30]. Gardeners continue to share the saved seeds for few crops not available in the market and with some cultural value (such as landraces), but they are less inclined to share seeds obtained in the market, as they expect other gardeners to also obtain those seeds through the market.

Low density of exchanges probably relates to the second commonality of the networks studied, fragmentation, although fragmentation does not fully explain low density. Our ethnographic understanding suggests that, in addition to low density, two more factors might help explain fragmentation: social distance between gardeners and physical distance between gardens. Researchers have highlighted that cultural distance between farmers (i.e., linguistic differences) prevent germplasm exchanges by increasing the costs of acquiring information [25,44,45]. Similarly, in our study areas, sociocultural differences between elderly who have traditionally lived in the areas and young migrants recently settled might help explain fragmentation in germplasm exchange networks. We have observed that the agricultural practices and crop preferences of the two groups often differ, preventing exchanges between those groups and thus contributing to network fragmentation. Additionally, we have also observed that in those areas where gardens are physically separated (i.e., Llanars, Llançà, Maçanet de Cabrenys, Molló in the Catalan Pyrenees), there are less exchanges than in those areas where gardens are closer to each other (i.e., Montejo de la Sierra or Valdemanco), the exception being the two valleys in our sample (Vall Fosca and Valle del Cordal), where -despite distance between gardens- there is not a high degree of fragmentation.

A third commonality between the networks analyzed is their low level of centralization. None of the networks in our sample includes a gardener or a group of them centralizing the exchange of seeds, not even in the case where one of the actors is a local seed bank (see [30] for a thorough analysis). Rather, germplasm seems to freely flow across the different gardeners who compose a subnetwork or component. A decentralized system of germplasm exchange could make networks of germplasm exchange more resilient to failures of a specific gardener (i.e., death, migration), since each gardener could potentially access multiple sources of germplasm providers [46,47]. This property, however, is conditional to the fact that the flows between gardeners are sufficient, timely, and accessible enough to re-stock seed lots if there were a loss.

We also found an important difference in the structure of the networks analyzed in relation to the presence of external links: in four of the 10 networks all the exchanges occurred among gardeners within the village, whereas in the other six networks there is an important prevalence of seed acquisition from individuals and organizations outside the village. We do not have a clear explanation for the differences found in our sample, although we think they might relate to the number of temporary and permanent migrants in each case study. For example, with the exception of Canencia, communities in the Sierra Norte, have several external actors. This is also the case in Asturias. In fact, many of the residents of those communities commute every day for work, or live in one community but have their gardens in another one. Those movements allow for fluid relations with people outside the studied network. In contrast, gardens in the Catalan Pyrenees (except in Vall Fosca) are mostly managed by elders who do not have the same social mobility than in the previous case, and who might –consequently have less external links. This characteristic of the networks could have implications on the overall network diversity as previous work suggests that plant propagation material acquired from the community is mostly oriented to regular supply of seeds, whereas the acquisition of germplasm from outside the community contributes to the introduction of new diversity [24,28,48] as the presence of external links might provide more choices.

### Agroecological knowledge and centrality

We now turn the discussion to the second significant finding of this paper: the association between a person’s centrality in the germplasm exchange network and his/her level of agroecological knowledge.

One of the two centrality measures tested, *broker*, bear a positive and statistically significant association with a person’s level of agroecological knowledge. This association easily finds explanation on previous literature. On the one side, the social network literature has also highlighted the preferential access to information for central actors in a network [12]. In his seminal work, Burt [49] borrowed the term *tertius gaudens* (“rejoicing third”) from the German sociologist Simmel to emphasize the ability of the *tertius* to pass accurate, ambiguous, or distorted information between otherwise unconnected nodes, thus highlighting the role of *brokers* in information exchange. On the other side, the germplasm exchange network literature has highlighted that seeds are not exchanged in a cultural vacuum; rather seeds often circulate accompanied with information related to their qualities and to particular management practices [25,50,51], which suggest an overlap in the network of seeds and information exchange. As we have seen in the field, germplasm exchange is always accompanied by detailed information about how to grow, care, and use the exchanged crop. Furthermore, we have often observed follow up questioning about the success of the exchanged crop, a situation that is used to reinforce the information given at the moment of exchange, or to add new information on the exchanged variety, or of gardening in general. Thus, because the physical exchange of germplasm contributes to create and strengthen social links, it is not surprising that we find a positive association between the position of a person on the network of germplasm exchanges and her level of agroecological knowledge.

But then, why only one of our two measures of centrality (i.e., *brokers*) is associated with agroecological knowledge? Why does not the variable weighted *degree* bear the same association? Knowledge is generated and transmitted through a complex process that includes more than two nodes (in our case, people who exchange seeds). Differently from the measure *degree* that captures the number of direct contacts between people, the measure “*broker”* also captures the person’s ability for bridging structural holes. Considering that planting material often flows with associated knowledge, people with a higher centrality, as measured by the variable *broker,* might hold a structural position that allows them to gain access to many pieces of group specific information (i.e., information shared by elders permanently living in the area, information brought by young newcomers, or information from clusters outside the village). Contrary, people with a large number of direct links, but not so well connected in the overall structure of the network, might be less aware of different alternatives. Access to preferential information across groups help explain the positive association between the measure *broker* in the network of germplasm exchange and our measure of agroecological knowledge.

## Conclusion

We conclude highlighting a theoretical and a policy implication of our study. At a theoretical level, our study emphasizes the role of the structural position of a person in a network in explaining intracultural variation on levels of traditional ecological knowledge. As other type of information, traditional ecological knowledge is embedded in social networks and may only be apparent in the context of relations and interactions. The finding that the centrality of an individual in a network helps explaining her/his level of traditional ecological knowledge highlights the importance of social relations in the construction of traditional ecological knowledge.

Our study has also a policy implication. Researchers have noticed the link between exchange of germplasm and *in situ* agrobiodiversity conservation [26,27,52], but have rarely studied the structure of the networks where those exchanges happen. Understanding the characteristics of germplasm exchange networks might help in the design of policies to sustain *in situ* agrobiodiversity. For example, our results suggest that, the studied networks display some common structural characteristics that could reduce the flow of planting material: low density of exchanges and network fragmentation. Taken together, the two characteristics imply that there are large proportions of gardeners that can not reach one to each other, thus reducing the number of possible exchanges. The networks also displayed some characteristics that –under certain circumstances- could enhance the flow of planting material: low centralization and the presence of external links. Policies that aim at sustaining *in situ* agrobiodiversity by increasing exchanges of planting material should focus on increasing the density of exchanges within the existing networks and on bridging relations between unconnected subgroups.

## Competing interests

The authors declare that they have no competing interests.

## Authors’ contributions

LC-M, LA-M, TG, JL, RO, MP, MP-S, MR and JV participated in the design of the study and in data collection. VR-G, JM, LC-M, carried out the statistical analysis. TG and VR-G coordinated the study. VR-G wrote the first draft of the manuscript. All authors read and approved the final manuscript.
